# Dr. Luman P. L. Parker

**Published:** 1899-02

**Authors:** 


Dr. Luman P. L. Parker, of Akron, N. Y., died Sunday evening, January 1, 1899, at his home in that village. Dr. Parker was born in Varysburg, Wyoming County, in 1828, consequently was 70 years old. His early life was spent upon a farm, after which he took a course in medicine in the University of Buffalo from which institution he was graduated in 1854. He was united in marriage to Miss Harriet Austin in 1850 and soon afterward located at Akron, where he had resided ever since. Four years ago he found it necessary to abandon his large practice on account of ill-health and had been confined to his home ever since. He is survived by four sons and one daughter, Edward E., of Austin, Pa.; William W., of Batavia; Charles C., Dr. Frank W. and Miss Harriet, of Akron.

